# Effectiveness and Safety of Intradetrusor OnabotulinumtoxinA Injection for Neurogenic Detrusor Overactivity and Overactive Bladder Patients in Taiwan—A Phase IV Prospective, Interventional, Multiple-Center Study (Restore Study)

**DOI:** 10.3390/toxins13120911

**Published:** 2021-12-20

**Authors:** Chung-Cheng Wang, Eric Chieh-Lung Chou, Yao-Chi Chuang, Chih-Chieh Lin, Yu-Chao Hsu, Chun-Hou Liao, Hann-Chorng Kuo

**Affiliations:** 1 Department of Urology, En Chu Kong Hospital, New Taipei City 237414, Taiwan; 00512@km.eck.org.tw; 2 Department of Biomedical Engineering, Chung Yuan Christian University, Chungli 320314, Taiwan; 3 Department of Urology, China Medical University Hospital, School of Medicine, China Medical University, Taichung 406040, Taiwan; d6573@mail.cmuh.org.tw; 4 Department of Urology, Kaohsiung Chang Gung Memorial Hospital, Kaohsiung 833401, Taiwan; chuang8270@adm.cgmh.org.tw; 5 Shu-Tien Urological Research Center, Department of Urology, Taipei Veterans General Hospital, School of Medicine, College of Medicine, National Yang Ming Chiao Tung University, Taipei 112304, Taiwan; cclin11@vghtpe.gov.TW; 6 Department of Urology, Linko Chang Gung Memorial Hospital, College of Medicine, Chang Gung University, Taoyung 333323, Taiwan; hsuyc@cgmh.org.tw; 7 Department of Urology, Cardinal Tien Hospital, School of Medicine, Fu-Jen Catholic University, New Taipei City 242062, Taiwan; 065294@mail.fju.edu.tw; 8 Department of Urology, Hualien Tzu Chi Hospital, Buddhist Tzu Chi Medical Foundation, Buddhist Tzu Chi University, Hualiang 970374, Taiwan

**Keywords:** overactive bladder, onabotulinum toxin A, neurogenic detrusor overactivity

## Abstract

We conducted a phase IV, pre/post multi-center study to evaluate the efficacy and safety of intradetrusor onabotulinumtoxinA injection in patients with neurogenic detrusor overactivity (NDO, *n* = 119) or overactive bladder (OAB, *n* = 215). Patients received either 200U (i.e., NDO) and 100U (i.e., OAB) of onabotulinumtoxinA injection into the bladder, respectively. The primary endpoint for all patients was the change in the PPBC questionnaire score at week 4 and week 12 post-treatment compared with baseline. The secondary endpoints were the changes in subjective measures (i.e., questionnaires: NBSS for patients with NDO and OABSS for those with OAB) at week 4 and week 12 post-treatment compared with baseline. Adverse events included symptomatic UTI, de novo AUR, gross hematuria and PVR > 350mL were recorded. The results showed that compared with baseline, PPBC (3.4 versus 2.4 and 2.1, *p* < 0.001) and NBSS (35.4 versus 20.4 and 18.1, *p* < 0.001) were significantly improved at 4 weeks and 12 weeks in NDO patients. In addition, compared with baseline, PPBC (3.5 versus 2.3 and 2.0, *p* < 0.001) and OABSS (9.1 versus 6.2 and 5.7, *p* < 0.001) were significantly improved at 4 weeks and 12 weeks in OAB patients. Eight (6.7%) had symptomatic UTI and 5 (4.2%) had de novo AUR in NDO patients. Twenty (9.3%) had symptomatic UTI but no de novo AUR in OAB patients. In conclusion, we found that intradetrusor onabotulinumtoxinA injections were safe and improved subjective measures related to NDO or OAB in our cohort.

## 1. Introduction

Overactive bladder (OAB) is defined as urinary urgency, commonly accompanied by frequent urination and nocturia, with or without urgency urinary incontinence (UUI) and without urinary tract infection (UTI) or any pathological condition [1]. An epidemiological survey in Asia showed that 16.9~20.8% of the population over 40 years old had OAB, which reduced many patients’ quality of life (QOL) and increased their health care expenses [2,3].

Neurogenic detrusor overactivity (NDO) can only be detected during urodynamic studies in individuals with an underlying neurological disorder, such as spinal bifida, spinal cord injury (SCI), stroke, or multiple sclerosis. Patients with NDO usually suffer from urine leaking during the storage phase because of decreased bladder capacity, decreased bladder compliance and increased intravesical pressure. These disease-associated changes can impact on the QOL and life expectancy of patients [4].

The primary treatment of OAB is behavioral modification or physical therapy, followed by oral medication (anti-muscarinic drugs and/or beta-3 adrenergic agonist) [5]. However, oral medications fail to provide effective treatment and have poor long-term tolerance [6,7].

In 2000, Schurch, et al., successfully injected onabotulinumtoxinA in patients with SCI and NDO for the first time [8]. Several large, randomized, placebo-controlled, phase III clinical studies showed that compared with the control group, injection of 200 units of onabotulinumtoxinA into the bladder could effectively reduce the number of UUI at week 6, increase bladder capacity and achieve significantly different levels of satisfaction in patient treatment [9]. Considering the adverse events, the onabotulinumtoxinA group had a higher chance of developing residual urine that required intermittent catheterization (relative risk (RR): 5.58, 95% confidence interval (C.I.): 3.53–8.83; *p* < 0.001) than the control group and higher chances of UTI (RR: 1.47, 95% CI: 1.29–1.67; *p* < 0.001), more hematuria (RR: 1.70, 95% CI: 1.01–2.85; *p* = 0.05) and muscle weakness (RR: 2.59, 95% CI: 1.36–4.91; *p* = 0.004).

In terms of treating OAB by injecting onabotulinumtoxinA, three large, randomized, placebo-controlled, phase III clinical studies have shown that the group injected with 100 units of onabotulinumtoxinA had a reduced number of episodes of UUI, improved QOL, increased patient confidence and interpersonal relationships and reduced social embarrassment and mental stress compared with the control group [10,11,12]. Considering safety, the injection group had a higher chance of developing uncomplicated UTIs (RR: 2.73; 95% CI: 1.98–3.78; *p* < 0.001), 93.9% of which did not require catheterization, 3.6% of which had less than 6 weeks of intermittent catheterization, 1.1% of which had 6 to 12 weeks of intermittent catheterization and 1.8% of which had over 12 weeks of intermittent catheterization.

In 2014, the Taiwan Food and Drug Administration approved the use of onabotulinumtoxinA in treating NDO and idiopathic OAB. In 2015, the benefits package of Taiwan’s National Health Insurance (NHI) Administration included intradetrusor onabotulinumtoxinA injections for refractory NDO and OAB. Thus, more patients were able to benefit from the health policy and were willing to receive intradetrusor therapy. Thus, the aim of the phase IV, pre/post interventional study was to conduct a close-to-real-world observation of the efficacy and safety of onabotulinumtoxinA, with the hope of constructing a post-marketing databank of onabotulinumtoxinA treatment outcomes in Taiwan.

## 2. Results

### 2.1. Therapeutic Effectiveness and Adverse Events in NDO Patients

The mean age of 119 NDO patients (72 men and 47 women) was 46.0 ± 16.1 (ranged 20 to 85) years. Thoracic SCI was the most common etiology of NDO (Table 1). A total of 72 (60.5%) of patients used clean intermittent self-catheterization, which was the most common method of current bladder management. Prior to treatment, 101 (84.9%) patients were refractory to oral medication. Post-treatment, Patient Perception Bladder Condition (PPBC), four domains of Neurogenic Bladder Symptom Score (NBSS) and QOL were significantly improved at 4 weeks and 12 weeks compared with baseline (Table 2). The therapeutic effect persisted since no differences in NBSS and PPBC were noted between the 4-week and 12-week follow-ups. However, PVR was significantly increased at 4 weeks and 12 weeks (Table 2). A total of eight (6.7%) patients had symptomatic UTIs, and five (4.2%) patients had de novo AURs during the 12-week follow-up. No gross hematuria was noted.

### 2.2. Therapeutic Effectiveness and Adverse Events in OAB Patients

The median age (interquartile range) was 68 (78–57) years in OAB patients (62 men and 153 female). The mean time since onset of OAB was 4.3 ± 5.1 years. The mean BMI was 24.9 ± 4.2. Prior to treatment, 153 (71.2%) patients were refractory to oral medication, 31 (14.4%) patients could not tolerate the side effects and 31 (14.4%) had both. These OAB patients could void spontaneously without any catheter. Table 3 shows that compared with baseline, PPBC and OAB Symptom Score (OABSS) were significantly improved at 4 weeks and 12 weeks. The differences in PPBC and OABSS between 4 weeks and 12 weeks were not statistically significant. Compared with baseline, PVR was significantly increased at 4 weeks and 12 weeks. However, the PVR at 12 weeks was significantly decreased compared with that at 4 weeks. A total of 20 (9.4%) patients had symptomatic UTI, but no de novo AUR or PVR > 350 mL was noted during the 12-week follow-up.

## 3. Discussion

To our knowledge, this is the first study reporting the efficacy and safety of intradetrusor onabotulinumtoxinA injection for the treatment of NDO and OAB patients in a postmarketing survey setting in Taiwan. We demonstrated that patients reporting outcomes in both NDO and OAB patients could be significantly improved after injection of 200 or 100 units of onabotulinumtoxinA, respectively. Although PVR was increased post-treatment, the de novo AUR in NDO patients was low and no OAB patients had de novo AUR. In addition, the study proved that inclusion of intradetrusor onabotulinumtoxinA injection in the benefits package of Taiwan’s NHI administration for NDO and OAB patients was a correct health strategy.

Recently, Walter et al., from Canada reported a phase IV clinical trial result and disclosed a beneficial effect of intradetrusor 200 units of onabotulinumtoxinA injections to reduce autonomic dysreflexia in patients with chronic SCI at T6 or above [13]. The main purpose of the phase IV postmarketing surveillance trial was to assure and monitor the efficacy and safety of the treatment, which might be compatible with or add some new findings to previous phase III clinical trials. In 2013, Rovner et al. summarized two double-blind, placebo-controlled, pivotal phase III trials including a total of 691 patients with at least 14 UUI episodes/week due to multiple sclerosis (MS) or SCI [14]. Compared with placebo groups, patients who received 200 units of intradetrusor onabotulinumtoxinA injection had significant improvements in UUI episodes, maximum cystometric capacity, maximum detrusor pressure during the first involuntary detrusor contraction and health-related QOL. Regarding adverse events, 51.8% of patients had UTIs and 19.9% had AURs. Using different evaluation methods, we showed that after intradetrusor injection of 200 units of onabotulinumtoxinA, patients with NDO had improvements in PPBC questionnaire and four domains in the NBSS questionnaire. Interestingly, the incidence rates of symptomatic UTI (6.7%) and de novo AUR (4.2%) in the study were lower than in previous phase III trials. This discrepancy might be explained by two different cohorts and different initial clinical presentations.

In treating refractory OAB patients with 100 units of intradetrusor onabotulinumtoxinA injection, our study had similar efficacy findings compared with previous large phase III trials. In 2013, two study groups from the United States and the United Kingdom demonstrated that compared with placebo, onabotulinumtoxinA significantly decreased UUI episodes per day and patients reporting OAB symptoms. In addition, onabotulinumtoxinA significantly improved patient health-related QOL. After injection, AUR was noted in 5.4% to 6.9% of patients [10,11]. In 2020, Yokoyama et al. from Japan showed that a significantly greater reduction was noted from baseline in daily UUI episodes in the onabotulinumtoxinA group than in the placebo group [12]. In the onabotulinumtoxinA group, there was a greater increase from baseline in the volume voided per micturition and greater improvement in patient reporting outcomes using King’s Health Questionnaire (KHQ) and OABSS questionnaires compared with the placebo group. After injection, de novo AUR and UTI were found in 6% and 13% of patients, respectively. However, in our series, symptomatic UTI was noted in only 4.6% of OAB patients, and no de novo AUR or PVR > 350 mL was found.

We first used NBSS, a comprehensive patient-reported outcome measurement specific for SCI, MS and spinal bifida patients with NDO in Asia. There were a total of 24 questions that covered current bladder management, incontinence, storage and voiding symptoms, urinary consequence complications and urinary-specific QOL associated with neurogenic bladder dysfunction. The validity and reliability of the NBSS were first reported in 2014, and the NBSS has been gradually used in several neurogenic bladder studies [15]. Using NBSS, Myers, et al., compared the effect of augmentation cystoplasty and botulinum toxin injection on patient-reported outcomes in SCI patients performing clean intermittent catheterization [16]. They found that augmentation cystoplasty was associated with better urinary function and satisfaction with urinary symptoms. In addition, Welk B., et al., demonstrated the responsiveness (i.e., the ability of a questionnaire to detect meaningful change) of the NBSS in SCI or MS patients receiving intradetrusor onabotulinumtoxinA injection [17]. The major findings were that the total NBSS score, the three subdomains of the NBSS and the urinary QOL question were responsive to positive clinical change and could be used to determine if patients significantly improved over time or after an intervention. In the study, all participants could easily answer the 24 questions within 10 min. Thus, we recommend the NBSS in the evaluation of neurogenic bladder patients in the future.

Taiwan’s NHI administration did not reimburse the use of intradetrusor onabotuliumtoxinA injection until 2015. Before that, patients paid at least 600 US dollars for one injection. Many low-income SCI patients may be particularly vulnerable to the underuse of essential drugs [18]. After 2015, Taiwan’s NHI administration provided almost complete drug coverage to beneficiaries who subsequently paid out-of-pocket costs of less than 20 US dollars for one injection. In addition, a previous study showed that onabotulinumtoxinA injection was cost-effective compared to anticholinergic medications for the treatment of refractory urge incontinence [19]. Combined with the evidence from our study that intradetrusor onabotulinumtoxinA injection was an effective and safe therapy for refractory NDO and OAB patients, the health strategy is a win-win solution for these patients.

Our study had several limitations. First, we only screened patients with voiding diaries, which were commonly used in phase III studies, but did not use voiding diary for follow-up. However, these validated questionnaires, including the PPBC, NBSS and OABSS, have been widely accepted as measurement tools for evaluating NDO and OAB patients. Secondly, the period of follow-up of patients was short (only 12 weeks). The regulation of Taiwan’s National Health Insurance Administration allowed repeated intradetrusor onabotulinumtoxinA injection if patients had no response to treatment at 3 months. The follow-up period was limited to 3 months to avoid evaluation of different injection time-points. Thus, the design of the study was to investigate the short-term efficacy and safety of onabotulinumtoxinA injection. Finally, all patients were refractory to behavioral therapy and oral medications before onabotulinumtoxinA treatment. The exact therapeutic results of treatment-naïve NDO and OAB patients are still unknown.

## 4. Conclusions

Our study demonstrated that intradetrusor onabotulinumtoxinA injection for NDO and OAB patients was effective and safe during a 12-week follow-up in a real-world setting in Taiwan.

## 5. Materials and Methods

The prospective study complies with the principles outlined in the declaration of Helsinki and was approved by the Ethics Committee of all participated hospitals (ECKIRB1040305). All of the patients were informed of the possible adverse events that might occur with onabotulinumtoxinA injection, and written informed consent forms with guarantees of confidentiality were obtained from the participants prior to treatment.

The aim of this study was to assess the effectiveness and safety of onabotulinumtoxinA injection into the bladder in treating NDO and OAB patients in postmarketing settings. From 2017 to 2019, 119 patients with NDO and 215 patients with OAB were recruited from six study hospitals in Taiwan. The inclusion criteria of NDO were adult (≥20 years old) urodynamically proven NDO patients with ≥14 episodes of UUI in one week. The inclusion criteria of OAB were adult patients (≥20 years old) with OAB symptoms with ≥14 episodes of UUI in one week. These NDO and OAB patients should have been taking antimuscarinic agents or beta-3 agonists for at least 3 months, with lack a response, or they were found to be intolerant to drug treatment. These inclusion criteria conform to the rules of Taiwan’s NHI Administration. All participants were treatment-naïve to intradetrusor onabotulinumtoxinA injection. In addition, patients should have sufficient cognitive and language abilities to complete questionnaires and should be willing to sign the informed consent forms. Before treatment, we educated all patients how to perform self-catheterization in case of AUR. Finally, a professional attending physician evaluated whether it was appropriate for the patients to receive onabotulinumtoxinA injection.

The main exclusion criteria were patient conditions that significantly interfered with the study as assessed by the attending physician, such as insufficient linguistic communication ability, inability to clearly understand the content of the informed consent form, refusal to receive education on intermittent catheterization and any unfit criteria as determined subjectively by the attending physician. In addition, patients with a history of allergies to the study drug onabotulinumtoxinA, participation in any study involving clinical drugs or medical devices in the last 90 days, allergies to anesthetics (such as lidocaine) used in the study and diagnoses of neuromuscular diseases, including myasthenia gravis, Eaton–Lambert syndrome, amyotrophic lateral sclerosis and planned invasive surgery, were all excluded.

This interventional study was approximately 14 weeks in duration and consisted of six visits. Two weeks before injection, the physician assessed and screened suitable subjects and explained the entire study process. Since all NDO and OAB patients were refractory to antimuscarinic agents or beta-3 agonist or intolerant to oral medication, patients were asked to stop taking antimuscarinic agents or beta-3 agonist and remained off them for the duration of the study. After the oral medication-free one-week period, patients could choose to resume oral medication or receive intradetrusor onabotulinumtoxinA injection. Patients with clinically suspected NDO underwent a urodynamic test. Only patients who had been proved NDO by urodynamic study could receive intradetrusor onabotulinumtoxinA injection. One week before injection, we obtained an informed consent form, assessed patients’ willingness for intermittent catheterization and evaluated their questionnaires. On the day of injection, patients with NDO received 200 units of onabotulinumtoxinA (Botox^®^, Allergan, Irvine, CA, USA) injection into the detrusor at 20 points; patients with OAB received 100 units of onabotulinumtoxinA injection into the detrusor at 10 points.

The subjective efficacy was evaluated using the NBSS and PPBC questionnaires in NDO patients and the OABSS and PPBC questionnaires in OAB patients at baseline, 4 and 12 weeks after treatment. The NBSS is a 24-item questionnaire that has three main domains (urinary incontinence, bladder storage and emptying and consequences), a non-scored question about bladder management and a single quality of life question [15]. The PPBC is commonly used to measure the incontinence severity and treatment outcome of OAB [20]. The OABSS, the sum score of four symptoms (daytime frequency, nighttime frequency, urgency and urgency incontinence), has been widely used for evaluation of OAB patients in many clinical study [21]. We did not use a voiding diary, a very important tool to evaluate the efficacy because the previous study has shown that the OABSS is highly sensitive to treatment-related changes of OAB symptoms. The OABSS can be an alternative to a bladder diary for symptom and efficacy assessment in daily clinical practice because of its simplicity and dependability [21].

Safety assessments were performed 1, 4 and 12 weeks after injection for adverse events and to measure PVR. The common adverse events included de novo AUR, symptomatic UTI, PVR volume >350 mL and gross hematuria with necessitating the use of an indwelling catheter.

The primary endpoint for all patients was the change in the PPBC questionnaire score at week 12 after injection compared with baseline. The secondary endpoint of NDO patients was the change in NBSS questionnaires at week 4 and week 12 after injection compared with baseline. The secondary endpoint of OAB patients was the change in OABSS questionnaires at week 4 and week 12 after injection compared with baseline.

Continuous normally distributed variables were reported as the mean value ± standard deviation (SD). Continuous non-normally distributed variables were presented as the median values and an interquartile range (IQR). Categorical data were expressed as numbers and percentages. The mean values of the continuous variables were compared using the Mann–Whitney U test. Categorical variables were compared using Fisher’s exact test. The differences between baseline, 4 weeks and 12 weeks were analyzed by one-way ANOVA and post hoc analysis. A *p* value of <0.05 was considered to be statistically significant. The incidence, severity and possible connection of adverse events were recorded and analyzed.

## Figures and Tables

**Table 1:** General characteristics of neurogenic detrusor overactivity patients.

NDO	*n* = 119
M:F	72:47
Age (years)	46.0 ± 16.1
Duration of disease (years)	10.0 ± 8.5
BMI	23.3 ± 3.0
Indication of BoNT_A injection	
No response to medication (%)	101 (84.9)
Side effects to medication (%)	12 (10.1)
Both (%)	6 (5.0)
Underlying neurological disorders	
C-SCI (%)	35 (29.4)
T-SCI (%)	45 (37.8)
L-SCI (%)	12 (10.1)
Spinal bifida (%)	6 (5.0)
Multiple sclerosis (%)	4 (3.4)
Cervical cancer after RH (%)	4 (3.4)
Spinal infection (%)	3 (2.5)
Spinal ganglioma (%)	3 (2.5)
Pelvic fracture (%)	3 (2.5)
Iatrogenic (%)	2 (1.7)
Spinal AVM (%)	2 (1.7)
Current bladder management	
Transurethral or suprapubic catheter (%)	13 (10.9)
Condom catheter (%)	2 (1.7)
CISC (%)	72 (60.5)
Spontaneous voiding (%)	28 (23.5)
CISC + spontaneous voiding (%)	4 (3.4)

NDO: neurogenic detrusor overactivity; OAB: overactive bladder; M: male; F: female; BMI: body mass index; BoNT-A: onabotulinumtoxinA; C-SCI: cervical spinal cord injury; T-SCI: thoracic spinal cord injury; L-SCI: lumbar spinal cord injury; RH: radical hysterectomy; AVM: arteriovenous malformation; CISC: clean intermittent self-catheterization.

**Table 2:** Comparison of the therapeutic effects and adverse events of neurogenic detrusor overactivity patients.

NDO	BL	1w	4w	12w	4w vs. 12w
Patient number	119	119	115	117	
PVR (mL)	187 ± 151	294 ± 183 *	300 ± 194 *	288 ± 181 *	0.68
PPBC	3.4 ± 0.8	-	2.4 ± 0.8 *	2.1 ± 1.0 *	0.06
NBSS-incontinence	10.4 ± 7.0	-	7.0 ± 6.0 *	6.0 ± 5.6 *	0.04
NBSS-voiding	8.4 ± 4.8	-	6.1 ± 4.3 *	5.6 ± 4.4 *	0.19
NBSS-consequence	6.8 ± 2.8	-	5.0 ± 2.7 *	4.5 ± 2.6 *	0.11
NBSS-QOL	3.0 ± 0.9	-	2.3 ± 0.9 *	2 ± 1 *	0.06
Symptomatic UTI (%)		1 (0.8)	5 (4.3)	2 (1.7)	
PVR > 350mL (%)	12 (10.1)	46 (38.7)	54 (47.0)	50 (42.7)	0.6
de novo AUR (%)		2 (1.7)	2 (1.7)	1 (0.9)	

NDO: neurogenic detrusor overactivity; BL: baseline; w: week; PVR: postvoiding residual urine; NBSS: neurogenic bladder symptom score; QOL: quality of life; UTI: urinary tract infection; AUR: acute urinary retention; *: significantly different from baseline.

**Table 3:** Comparison of the therapeutic effects and adverse events of overactive bladder patients.

	BL	1w	4w	12w	4w vs. 12w
Patient number	215	215	191	212	
PPBC	3.5 ± 0.8	-	2.3 ± 1 *	2.0 ± 1.2 *	0.005
OABSS-1	1.6 ± 0.6	-	1.2 ± 0.5 *	1.1 ± 0.6 *	0.28
OABSS-2	2.2 ± 0.8	-	1.7 ± 0.8 *	1.5 ± 0.8 *	0.11
OABSS-3	3.1 ± 1.5	-	2.1 ± 1.4 *	1.9 ± 1.3 *	0.08
OABSS-4	2.2 ± 1.6	-	1.2 ± 1.3 *	1.1 ± 1.3 *	0.3
OABSS-total	9.1 ± 3.1	-	6.2 ± 2.7 *	5.7 ± 2.7 *	0.06
PVR (mL)	46 ± 52	86 ± 77 *	98 ± 88 *	79 ± 67 *	0.02
Symptomatic UTI (%)		7 (3.3)	7 (3.7)	6 (2.8)	
de novo AUR (%)		0	0	0	
PVR > 350mL (%)		0	0	0	

BL: baseline; w: week; PPBC: patient perception bladder condition; OABSS: overactive bladder symptom score; PVR: postvoiding residual urine; UTI: urinary tract infection; AUR: acute urinary retention; * significantly different from baseline.

## Data Availability

The data presented in this study are available on request from the corresponding author. The data are not publicly available due to privacy.

## Abbreviations

OAB	overactive bladder
NDO	neurogenic detrusor overactivity
QOL	quality of life
UTI	urinary tract infection
RR	relative risk
CI	confidence interval
NHI	national health insurance
PPBC	patient perception bladder condition
NBSS	neurogenic bladder symptom score
OABSS	overactive bladder symptom score
PVR	postvoiding residual urine
BMI	body mass index
SCI	spinal cord injury
MS	multiple sclerosis
AUR	acute urinary retention
